# PGC-1α activity and mitochondrial dysfunction in preterm infants

**DOI:** 10.3389/fphys.2022.997619

**Published:** 2022-09-26

**Authors:** Atefeh Mohammadi, Randa Higazy, Estelle B. Gauda

**Affiliations:** ^1^ The Hospital for Sick Children, Division of Neonatology, Department of Pediatrics and Translational Medicine Program, Toronto, ON, Canada; ^2^ Department of Laboratory Medicine and Pathobiology, University of Toronto, Toronto, ON, Canada

**Keywords:** mitochondrial dysfunction, oxidative stress, reactive oxygen species, PGC-1α, white matter injury, bronchopulmonary dysplasia

## Abstract

Extremely low gestational age neonates (ELGANs) are born in a relatively hyperoxic environment with weak antioxidant defenses, placing them at high risk for mitochondrial dysfunction affecting multiple organ systems including the nervous, respiratory, ocular, and gastrointestinal systems. The brain and lungs are highly affected by mitochondrial dysfunction and dysregulation in the neonate, causing white matter injury (WMI) and bronchopulmonary dysplasia (BPD), respectively. Adequate mitochondrial function is important in providing sufficient energy for organ development as it relates to alveolarization and axonal myelination and decreasing oxidative stress via reactive oxygen species (ROS) and reactive nitrogen species (RNS) detoxification. Peroxisome proliferator-activated receptor gamma coactivator-1 alpha (PGC-1α) is a master regulator of mitochondrial biogenesis and function. Since mitochondrial dysfunction is at the root of WMI and BPD pathobiology, exploring therapies that can regulate PGC-1α activity may be beneficial. This review article describes several promising therapeutic agents that can mitigate mitochondrial dysfunction through direct and indirect activation and upregulation of the PGC-1α pathway. Metformin, resveratrol, omega 3 fatty acids, montelukast, L-citrulline, and adiponectin are promising candidates that require further pre-clinical and clinical studies to understand their efficacy in decreasing the burden of disease from WMI and BPD in preterm infants.

## 1 Introduction

Extremely low gestational age neonates (ELGANs) born less than 28 weeks of gestation are at increased risk for a myriad of comorbidities including white matter injury (WMI) in the brain, bronchopulmonary dysplasia (BPD), necrotizing enterocolitis (NEC), and retinopathy of prematurity (Kelly, 2006; Thébaud et al., 2019). Apnea of prematurity is also common in these infants wherein periods of intermittent hypoxia are frequent (Kelly, 2006). Intermittent hypoxia increases reactive oxygen species (ROS) and reactive nitrogen species (RNS), leading to oxidative stress and mitochondrial dysfunction with adverse consequences to developing organs. In this review, we discuss the downstream impacts of oxidative stress and mitochondrial dysfunction leading to WMI and BPD, two major comorbidities in ELGANs. Moreover, we discuss the importance of peroxisome proliferator-activated receptor gamma coactivator-1 alpha (PGC-1α), master regulator of mitochondrial biogenesis and function, as it relates to the pathogenesis of WMI and BPD, and potential therapies that indirectly regulate PGC-1α activity to reduce the burden of disease in ELGANs.

## 2 Mitochondrial dysfunction in preterm infants and its downstream effects

Substantial cellular energy is required throughout gestation for organ development and growth. ELGANs have especially high energy needs for adequate postnatal organ development since they are born with structurally and functionally immature organs (Ten, 2017; Harvey, 2019; Rodríguez-Cano et al., 2020). Mitochondria are responsible for cellular energy generation in the form of adenosine triphosphate (ATP) by oxidative phosphorylation through the electron transport chain (ETC). The ETC is composed of five multi-subunit enzyme complexes (complex I-V) and two electron carriers (Coenzyme Q and cytochrome c) located in the inner mitochondrial membrane (Guo et al., 2013). The transfer of electrons through the ETC is coupled with the transport of protons across the inner membrane, establishing the electrochemical gradient which allows for the protonmotive force and ATP production (Guo et al., 2013). During this process, mitochondria continuously function to metabolize oxygen and generate ROS and RNS. Mitochondria are equipped with defense systems to detoxify ROS and RNS and protect the cell against oxidative damage. Some antioxidant enzymes are manganese superoxide dismutase (SOD2), catalase, thioredoxin reductase 2, peroxiredoxin (PRX)-5, PRX-3, uncoupling protein (UCP)-2, and thioredoxin (TRX)-2 (MatÉs et al., 1999; Mailloux, 2018; Napolitano et al., 2021).

**FIGURE 1:**
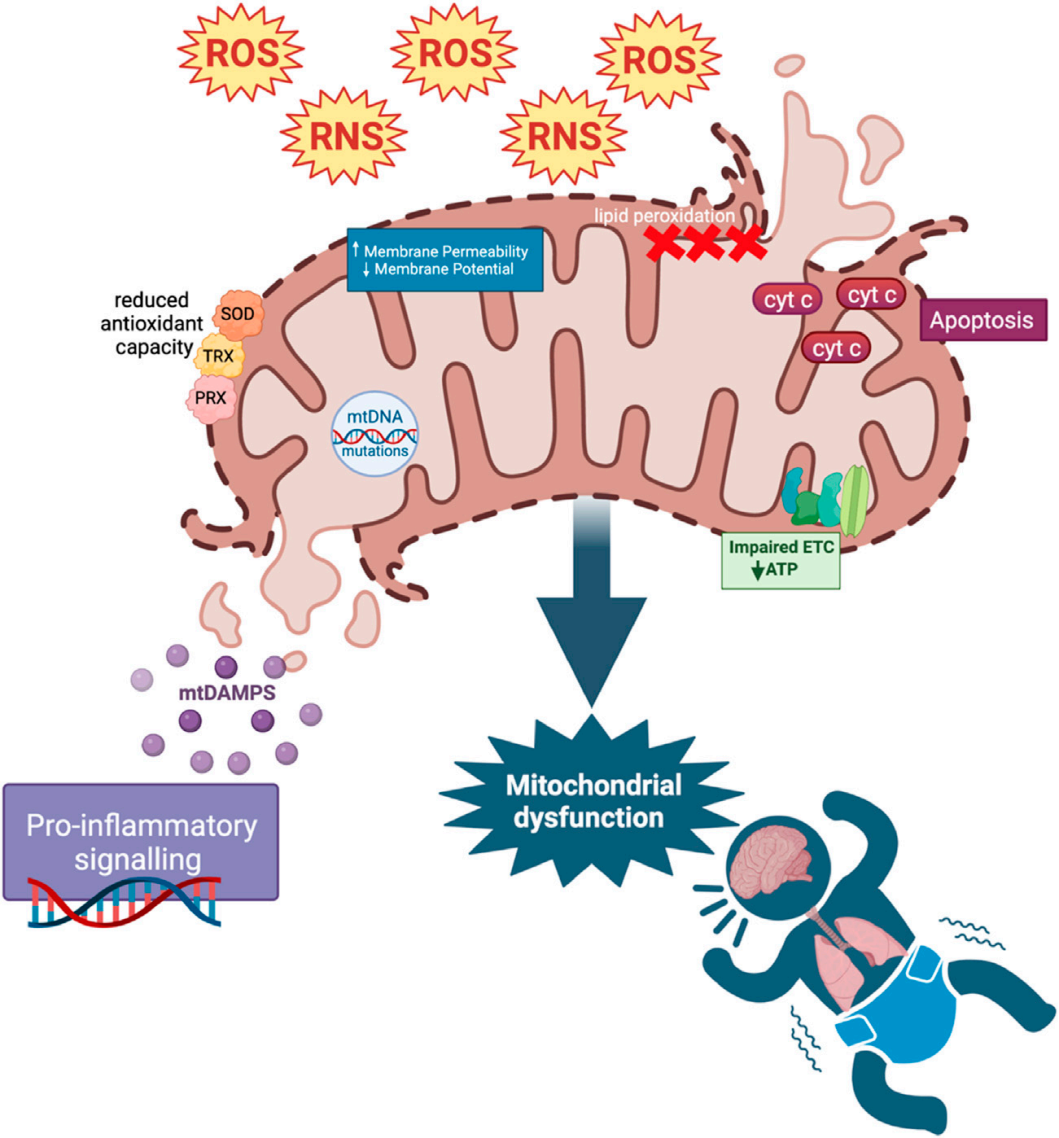
ROS and Mitochondrial dysfunction. Increased ROS and RNS cause mitochondrial dysfunction through mitochondrial DNA (mtDNA) mutations, defects in the electron transport chain (ETC) and antioxidant modulatory proteins, and lipid peroxidation in cell and mitochondrial membranes (affecting mitochondrial membrane potential). These changes in mitochondrial permeability could lead to the release of cytochrome C (cyt c), mediating apoptosis as well as mitochondrial DAMP (mtDAMP) release. MtDAMPs and increased ROS cause downstream pro-inflammatory signaling. Reduced antioxidant capacity resulting from mitochondrial dysfunction further adds to the pool of ROS in a positive feedback loop.

Under normal physiological conditions, ROS and RNS levels are balanced by the enzymatic and non-enzymatic antioxidant detoxification systems (Douglas et al., 2010; Zhang et al., 2016). However, under pathologic conditions, ROS and RNS production exceeds detoxification, leading to oxidative and nitrosative stress (Mittal et al., 2014; Zhang et al., 2016; Cannavò et al., 2021). Elevated ROS and RNS levels cause injury *via* lipid peroxidation, cell death, and release of damage-associated molecular patterns (DAMPs), initiating an inflammatory response (Circu and Aw, 2010; Nakahira et al., 2015; Su et al., 2019; Falsaperla et al., 2020). ROS overproduction can cause pathophysiological changes in the mitochondria, known as mitochondrial dysfunction. It involves mitochondrial DNA mutations, ETC dysregulation, increased membrane permeability, and impaired antioxidant modulatory systems (Guo et al., 2013) (Figure 1). Damage to mitochondria can also lead to the release of cytotoxic mitochondrial DAMPs that can initiate innate and adaptive immune responses (Nakahira et al., 2015). Mitochondria have various mechanisms such as mitochondrial fusion and fission, biogenesis, and mitophagy to maintain homeostasis and prevent ROS-induced oxidative damage. Mitophagy is an autophagic response by which dysfunctional mitochondria are targeted for lysosomal degradation, preventing further ROS production and cytotoxicity (Wu et al., 2019). The accumulation of damaged mitochondria and a decrease in mitophagy are hallmarks of organ failure (Zhulong et al., 2021). Lastly, mitochondria continuously undergo fission and fusion to allow for the exchange or segregation of material between mitochondria to repair damaged components and to ensure energy needs are met (Thornton et al., 2018; Jones and Thornton, 2022).

At birth, preterm infants with decreased antioxidant capacities are exposed to a high oxidative load, creating substantial risk for cellular and tissue injury because the intrauterine environment is hypoxic relative to the extrauterine environment (Mittal et al., 2014; Ozsurekci and Aykac, 2016; Falsaperla et al., 2020; Cannavò et al., 2021). In this review, we discuss the role of mitochondrial dysfunction in the pathobiology of WMI and BPD.

### 2.1 Mitochondrial dysfunction in the brain

Preterm infants have structurally and functionally immature brains because major components of brain development including oligodendrocyte maturation, myelination, and synapse formation occur in the latter stages of gestation (Jakovcevski et al., 2009; Stiles and Jernigan, 2010; Moura et al., 2022). Adequate brain development requires increased total mitochondrial capacity and higher ATP levels to meet the significantly increased rate of energy consumption (Erecinska et al., 2004; Ten, 2017). Mitochondrial dysfunction is operative in injury to the developing oligodendrocyte, cells responsible for myelination, leading to WMI (Bizzozero et al., 1999; Morató et al., 2014). WMI is characterized as abnormal axonal myelination in the brain (Ten, 2017). Oligodendrocytes are highly sensitive to changes in mitochondrial function. Inhibition of mitochondrial complexes I and IV results in arrested oligodendrocyte differentiation and degeneration of mature and precursor cells, respectively. Impaired mitochondrial ETC activity, particularly at complexes I and III, also leads to increased ROS production, and subsequent neuronal cell death (Douglas et al., 2010). ROS levels are further increased as a result of impaired antioxidant defenses by enzymes such as SOD and glutathione peroxidase which are low in infants born 28 weeks of gestation (Flint Beal, 1995; Neubauer, 2001; Prabhakar et al., 2006; Shan et al., 2007; Lee and Davis, 2011; Lembo et al., 2021). When brain tissue is injured, it resorbs. Depending on the stage of brain development when the injury occurs, these infants can have poor brain growth leading to microcephaly or hydrocephalus ex vacuo (enlargement of the ventricles due to resorbed brain tissue). Periventricular leukomalacia (PVL) which involves the death of brain tissue around the ventricles exists in two forms, non-cystic and cystic PVL. Non-cystic PVL affects ∼50% of ELGANs whereas, the more severe form, cystic PVL, affects ∼5% of these infants (Khwaja and Volpe, 2008; Ten, 2017). Abnormal axonal myelination from arrested oligodendrocyte maturation is associated with poor motor outcomes, while injury to migrating neurons from the subventricular region to the cortex is associated with cognitive, behavioral, attentional, and sensorimotor deficits (Lee, 2017; Ten, 2017).

Breathing instability is a hallmark of the preterm infant due to the immature central respiratory network along with the strong influence of peripheral arterial chemoreceptors on baseline breathing. The combination of low functional residual capacity, ventilation-perfusion mismatch, and hypoxic pulmonary vasoconstriction, increases the risk of intermittent hypoxic episodes which are common and significant in ELGANs. Notably, both hypoxia and hyperoxia lead to ROS production (Chandel et al., 2000; Guzy and Schumacker, 2006), and repetitive episodes of intermittent hypoxia in preterm infants are associated with increased markers of oxidative stress, ROS generation, and mitochondrial dysfunction (Schulz et al., 2000; Lavie, 2003; Douglas et al., 2010). Early postnatal intermittent hypoxia in newborn rats causes WMI, hypomyelination, apoptosis, and an increase in pro-inflammatory markers in the brain (Gozal et al., 2001a; Gozal et al., 2001b; Xu et al., 2004; Douglas et al., 2010; Cai et al., 2012; Juliano et al., 2015; Darnall et al., 2017; Ten, 2017). Intermittent hypoxia in adult rodents also causes apoptosis, mitochondrial dysfunction, oxidative stress, and ROS generation in the brain (Gozal et al., 2001a; Gozal et al., 2001b; Row et al., 2003; Xu et al., 2004). Transgenic mice overexpressing the antioxidant enzyme extracellular SOD (SOD3) have lower levels of ROS and reduced neuronal apoptosis in response to hypoxia suggesting that upregulation of SOD in preterm infants at risk for WMI may be protective (Zaghloul et al., 2014).

Systemic inflammation is another factor that markedly increases the risk of developing neuroinflammation and subsequent WMI in ELGANs (Volpe, 2008; Lee, 2017). For instance, preterm infants with inflammatory diseases such as BPD and NEC are at increased risk of developing WMI than infants without BPD or NEC (Shah et al., 2008; Volpe, 2008; Gagliardi et al., 2009; Biouss et al., 2019; Agut et al., 2020; Grelli et al., 2021). Circulating cytokines, chemokines, and DAMPs originating from inflamed tissue can damage the central nervous system through the bloodstream and a disrupted blood-brain barrier. Increased blood-brain barrier permeability directly disrupts brain homeostasis (Sankowski et al., 2015; Kempuraj et al., 2017; Varatharaj and Galea, 2017; Galea, 2021). The influx of inflammatory mediators can trigger neuroinflammation and activate microglia; microglia are specialized macrophages in the central nervous system. Activated microglia produce increased levels of ROS and RNS that can damage developing neurons and cells that lead to WMI in preterm infants (Kadhim et al., 2006; Deng et al., 2008; Niizuma et al., 2010; Chen et al., 2011; Franklin, 2011; Baburamani et al., 2014; Back, 2017; Kempuraj et al., 2017; Lee et al., 2019; McNamara and Miron, 2020).

### 2.2 Mitochondrial dysfunction in the lungs

BPD is a chronic lung disease initiated by inflammation and oxidative stress during the canalicular and saccular stages of lung development, disrupting alveolar and vascular growth (Thébaud et al., 2019). Mitochondrial dysfunction, impaired oxidative phosphorylation, and their downstream effects are all operative in BPD pathogenesis (Ratner et al., 2009; Yue and Yao, 2016; Kandasamy et al., 2017; Shah et al., 2018; Xuefei et al., 2021; Yue et al., 2021). Mitochondrial density continues to increase during the postnatal period to support normal lung development and growth but mitochondrial density and function are disrupted in infants with BPD (El-Merhie et al., 2017). Preterm infants with BPD have increased ROS production and mitochondrial dysfunction which lead to impaired tissue development (Berkelhamer et al., 2013a; Collins et al., 2017; Ten, 2017; Valencia et al., 2018; Wang and Dong, 2018). Hyperoxia-induced mitochondrial DNA damage negatively impacts branching morphogenesis and lung development (Ballinger et al., 1999; Gebb et al., 2013). Mitochondrial dysfunction and inhibition of Complex I in neonatal mice results in disrupted alveolarization, a key feature of BPD, suggesting an important role for mitochondrial regulation and function in lung development (Bizzozero et al., 1999; Ratner et al., 2009; Vohwinkel et al., 2011; Das, 2013; Ratner et al., 2013). Mitochondrial dysfunction also occurs in adult lung diseases such as pulmonary fibrosis and chronic obstructive pulmonary disorder (Ahmad et al., 2015; Yue and Yao, 2016; Rangarajan et al., 2017; Cloonan et al., 2020; Larson-Casey et al., 2020; Siekacz et al., 2021).

The clinical definition of BPD is a need for supplemental oxygen at 36 weeks corrected gestational age in a formerly preterm infant (Jensen et al., 2019; Sahni and Mowes, 2022). Although the concentration of inhaled supplemental oxygen may not lead to toxic levels of oxygen in the blood, it is directly toxic to the bronchial and alveolar epithelial cells that line the airway and internal lung surface area. Oxygen toxicity in alveolar epithelial cells is characterized by abnormal mitochondrial morphology, impaired mitochondrial biogenesis, increased mitochondrial ROS production, and mitochondrial DNA damage (O’Donovan and Fernandes, 2000; Ahmad et al., 2001; Dobson et al., 2002; Roper et al., 2004; Ruchko et al., 2005; Brueckl et al., 2006; Chan, 2006; Ratner et al., 2009; Afolayan et al., 2012; Das, 2013; Yue and Yao, 2016; Kandasamy et al., 2017; Shah et al., 2018). Alveolar Type 1 epithelial cells cover 95–98% of the internal lung surface area and are vulnerable to oxygen toxicity even at low levels of inhaled oxygen (Harris et al., 1991; McElroy and Kasper, 2004). Mitochondrial density varies with cell type and function. For instance, alveolar epithelial Type 2 cells have a high number of mitochondria to support surfactant production (Massaro et al., 1975). Notably, hyperoxia-associated mitochondrial dysfunction is not rapidly reversible; mitochondria dysregulation persists in alveolar epithelial cells exposed to hyperoxia even after recovery in normoxic conditions (Garcia et al., 2021). Alveolar epithelial cells, specifically Type 1 cells, are in close proximity to endothelial cells as they play an important role in facilitating gas exchange. Human umbilical vein endothelial cells (HUVECs) are often used to study endothelial function. HUVECs from preterm infants who later developed BPD or died had increased indicators of mitochondrial dysfunction when the cells harvested at birth were exposed to hyperoxia *in vitro* (Kandasamy et al., 2017). Specifically, these cells produced more mitochondrial ROS, had a lower capacity for oxidative phosphorylation, decreased oxygen consumption, and more mitochondrial DNA damage (Kandasamy et al., 2017). These effects were not seen in HUVECs harvested from infants without BPD. Another characteristic feature of BPD is chronic hypercapnia; elevated carbon dioxide levels cause mitochondrial dysfunction as well as inhibition of oxidative phosphorylation, which decreases cell proliferation in alveolar epithelial cells and lung fibroblasts (Vohwinkel et al., 2011; Thébaud et al., 2019).

Pulmonary hypertension (PH) is a common comorbidity in preterm infants with BPD, occurring in approximately 25% of infants with moderate-severe BPD (Berkelhamer et al., 2013b; Mourani et al., 2015; Al-Ghanem et al., 2017; Weismann et al., 2017; Hansmann et al., 2021) with mortality rates of 14–38% in infants with BPD-PH (Khemani et al., 2007; An et al., 2010; Ambalavanan and Mourani, 2014; Al-Ghanem et al., 2017; Arjaans et al., 2021). PH is characterized by pulmonary artery remodeling and increased pulmonary vascular resistance, leading to right ventricular dysfunction (Berkelhamer et al., 2013b; Hansmann et al., 2021). Another feature of PH pathophysiology is pulmonary artery smooth muscle cell (PASMC) and endothelial cell hyperproliferation and resistance to apoptosis, causing pulmonary artery wall thickening and lumen narrowing (Marshall et al., 2018; Humbert et al., 2019; Nesterova et al., 2020). Mitochondrial dysfunction and dysregulation are also operative in PH pathogenesis (Trenker et al., 2007; Angara and Bhandari, 2013; Dromparis et al., 2013; Pak et al., 2013; Adesina et al., 2015; Bruns et al., 2015; Diebold et al., 2015; Houten, 2015; Chan and Rubin, 2017; Hong et al., 2017; Culley and Chan, 2018; Marshall et al., 2018; Rafikova et al., 2018; Suliman and Nozik-Grayck, 2019). Neonatal mice who developed hyperoxia-mediated BPD-PH with right ventricular hypertrophy had reduced expression of antioxidant enzymes and increased expression of ROS-producing enzymes in lung homogenates, compared to normoxic controls (Datta et al., 2015). The mechanisms by which hyperoxia, hypoxia, and inflammation contribute to an imbalance in ROS production and detoxification, oxidative stress, mitochondrial dysfunction, and characteristic PH pathophysiology in PASMCs and endothelial cells, are summarized by Marshall and colleagues (Iwata et al., 2014; Adesina et al., 2015; Rafikov et al., 2015; Ghouleh et al., 2017; Marshall et al., 2018; Martinho et al., 2020). Ultimately, mitochondrial dysregulation leads to mitophagy and decreased mitochondrial biogenesis, as described in animal models and patients with PH (Haslip et al., 2015; Ryan et al., 2015; Aggarwal et al., 2016; Marshall et al., 2018). Administration of mitoTEMPO, a mitochondrial-specific antioxidant, and activation of nuclear respiratory factor 2 (NRF-2), a transcription factor that reduces ROS production, improves mitochondrial dysfunction, decreases ROS levels, and attenuates the pathophysiology associated with BPD-PH in mice (Eba et al., 2013; Datta et al., 2015).

Collectively, the published data strongly support an important role for mitochondrial dysfunction in the pathogenesis of BPD-PH and its associated mortality. Thus, strategies that can preserve mitochondria integrity during early development in ELGANs could potentially be useful in reducing the burden of disease from BPD-PH.

## 3 The role of PGC-1α in mitochondrial dysfunction

Peroxisome proliferator-activated receptor gamma coactivator-1 alpha (PGC-1α) is a major transcriptional coactivator of several critical downstream pathways that regulate mitochondrial biogenesis, ROS detoxification, and oxidative phosphorylation (Wu et al., 1999; Villena, 2015). PGC-1α is also critical in regulating cellular metabolism, thus its expression is enhanced in tissues and organs with high energy requirements including the liver, cardiac and skeletal muscle, kidney, brown adipose tissue, brain, and retina (Handschin and Spiegelman, 2006; Liang and Ward, 2006; Handschin, 2009). Importantly, PGC-1α expression and activity are very tissue specific and while studies have examined tissues that highly express PGC-1α, our knowledge of the isoforms expressed in the brain and lungs and how they are regulated is incomplete (Martínez-Redondo et al., 2015; Jannig et al., 2022).

PGC-1α co-activates transcription factors involved in mitochondrial biogenesis and function, including nuclear respiratory factors (NRF−1 and −2), peroxisome proliferator-activated receptors (PPARs), mitochondrial transcription factor A (TFAM), and estrogen-related receptor alpha (ERRα) (Bastin et al., 2008; Rebelo et al., 2011; Gureev et al., 2019). PGC-1α decreases mitochondrial ROS by modulating the expression and activity of mitochondrial antioxidant enzymes including SOD2, catalase, thioredoxin reductase 2, PRX-5, PRX-3, UCP-2, and TRX-2 (Valle et al., 2005; Rius-Pérez et al., 2020). These free radical scavenging enzymes protect cells from mitochondrial dysfunction and subsequent oxidative stress.

PGC-1α dysregulation is also closely tied with inflammation and the nuclear factor kappa-light-chain-enhancer of activated B cells (NF-κB) signaling pathway (Valle et al., 2005; Handschin and Spiegelman, 2006; Palomer et al., 2009; Remels et al., 2013; Eisele et al., 2015; Dinulovic et al., 2016; Kadlec et al., 2016). PGC-1α directly inhibits NF-κB by binding to the p65 subunit and preventing its translocation to the nucleus and transcription of genes encoding pro-inflammatory cytokines (Alvarez-Guardia et al., 2010; Eisele et al., 2013). Inflammation represses PGC-1α activity which subsequently downregulates the gene expression of mitochondrial antioxidants, increases ROS levels and oxidative injury, and further exacerbates the inflammatory response. The relationship between PGC-1α, inflammation, and oxidative stress is summarized by Rius-Pérez and colleagues (Rius-Pérez et al., 2020).

### 3.1 PGC-1α dysregulation in the brain

Normal brain development has high energy requirements hence, mitochondria biogenesis and effective detoxification of ROS and RNS are essential to the process (Schon and Manfredi, 2003; Li et al., 2017). Studies using brain tissue from adults with multiple sclerosis, a demyelinating disease triggered by inflammation, show that a low level of PGC-1α is associated with the process of demyelination (Nijland et al., 2014). PGC-1α overexpression in human astrocytes reduced pro-inflammatory mediators, ROS production, and cell death, and protected neuronal cells co-cultured with the astrocytes against oxidative injury, suggesting a protective role for PGC-1α in reducing neurodegeneration (Nijland et al., 2014). These findings support the need for future studies exploring the role of PGC-1α in normal and abnormal brain development, specifically as it relates to WMI and PVL in preterm infants.

Kuczynska and colleagues have written a comprehensive review on the role of PGC-1α in the central and peripheral nervous system and its involvement in various neurodegenerative and neuropsychiatric disorders in adults (Schon and Manfredi, 2003; Chen et al., 2011; Kuczynska et al., 2022). Notably, PGC-1α deficiency in adults leads to abnormal myelination, neurodegeneration, and structural abnormalities seen in amyotrophic lateral sclerosis (ALS), Parkinson’s, Huntington’s, and Alzheimer’s diseases in adult humans and animal models (Lin et al., 2004; Leone et al., 2005; Zheng et al., 2010; Thau et al., 2012; Tsunemi et al., 2012; Johri et al., 2013; Lucas et al., 2015; Sweeney and Song, 2016; Mota and Sastre, 2021; Piccinin et al., 2021). These disorders are also associated with oxidative stress, mitochondrial dysfunction, and neuroinflammation. Microglia in the central nervous system have important immune functions and can display pro-inflammatory (M1) or anti-inflammatory (M2) phenotypes depending on the surrounding microenvironment (Gagliardi et al., 2009). They also play a large role in brain development, particularly in myelination (McNamara and Miron, 2020). During inflammation, PGC-1α is necessary for promoting and maintaining the M2 anti-inflammatory microglia phenotype (Yang et al., 2017). PGC-1α overexpression in microglia significantly decreases ischemic injury-induced neurologic deficits by reducing NLRP3 inflammasome activation and pro-inflammatory cytokine production, and enhancing mitophagy in adult mice (Han et al., 2021).

Given the role of mitochondrial dysfunction and oxidative stress in adult disease pathophysiology and the significance of PGC-1α as a master regulator of mitochondrial biogenesis, it is likely that PGC-1α signaling also plays an important role in the pathogenesis of WMI and PVL. However, there are limited preclinical models in newborn animals examining the role and importance of PGC-1α activity and regulation in neonatal brain injury. Additionally, emerging evidence suggests that hypoxia may also regulate PGC-1α activity (Shoag and Arany, 2010). Neonatal hypoxic-ischemic brain injury in rats induces mitochondrial biogenesis, specifically increasing NRF-1 and TFAM gene and protein expression (Yin et al., 2008). Intermittent hypoxia also has a neuroprotective effect in adult rats with cerebral ischemia injury, by improving mitochondrial health and function as well as promoting mitochondrial biogenesis through AMPK-PGC-1α-SIRT3 activation (Su et al., 2022). Further studies are required to understand the complex relationship between hypoxia and PGC-1α signaling and its relevance to neonates who experience periods of intermittent hypoxia is particularly critical.

### 3.2 PGC-1α dysregulation in the lungs

BPD is characterized by arrested lung development, and although limited, published data support an essential role for PPARγ and PGC-1α in numerous processes involved in lung development. For instance it stimulates interstitial lipofibroblast maturation and differentiation, and the transdifferentiation of myofibroblasts into lipofibroblasts, essential for alveolarization (Torday et al., 2003; Simon et al., 2006a; Simon et al., 2006b; Rehan et al., 2006; Rehan and Torday, 2012). PGC-1α also activates TFAM which is key for normal lung development and postnatal survival; TFAM mutations *in utero* lead to lethal abnormalities in branching morphogenesis (Srivillibhuthur et al., 2018). The Wnt/β-catenin pathways involved in organogenesis is tightly regulated during lung development, injury, and repair. This pathway is persistently activated in infants with BPD (Lingappan and Savani, 2020). Overactivation of the Wnt/β-catenin canonical pathway is associated with disrupted lung development and maturation (Lecarpentier et al., 2019; Zhang et al., 2020). Increased activation of this pathway has an inhibitory effect on PPARγ. The relationship between the Wnt/β-catenin and PPARγ pathways are reviewed in detail by Lecarpentier and colleagues (Lecarpentier et al., 2019).

**FIGURE 2:**
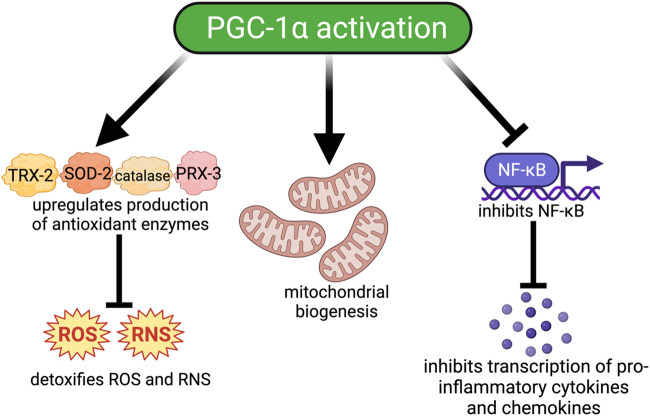
Downstream signaling pathways modulated by PGC-1α activation. Activation of PGC-1α. 1) improves oxidative injury by increasing the production of antioxidant enzymes such as catalase, thioredoxin-2 (TRX-2), peroxiredoxin-3 (PRX-3), and manganese superoxide dismutase (SOD-2) which detoxify ROS/RNS; 2) Increases mitochondrial biogenesis by upregulating the transcription of important transcription factors such as NRF-1, NRF-2, and TFAM; and 3) Reduces inflammation by inhibiting NF-κB and the transcription of proinflammatory cytokines and chemokines.

M2 macrophages in the lung are critical for branching morphogenesis and alveolarization during lung development (Jones et al., 2013). PGC-1α, similar to its role in the brain, regulates macrophage polarization to an anti-inflammatory M2 phenotype during inflammation (Eisele et al., 2013; Eisele et al., 2015; Fontecha-Barriuso et al., 2019). Moreover, aberrant PGC-1α regulation is found in tissue from patients with idiopathic pulmonary fibrosis, asthma, COPD, PH, and lung cancers (Li et al., 2010; Mata et al., 2012; Rao et al., 2012; Ye et al., 2016; Chan and Rubin, 2017; Caporarello et al., 2019; Aghapour et al., 2020; Bueno et al., 2020; Larson-Casey et al., 2020; Oh et al., 2021; Saito et al., 2021; Xu et al., 2022). Taken together these data from pre-clinical studies support an important and necessary role for PGC-1α in normal lung development and dysregulation. Fewer data are available describing the role of PGC-1α in BPD-PH. However, since PGC-1α regulates mitochondrial function, oxidative stress, and inflammation, all of which are essential processes involved in BPD-PH pathogenesis, it is highly likely that it could be an effective therapeutic target that could modify the development of BPD-PH (2, 71, 76). Thus, for the remainder of this review, we will discuss therapeutics that can upregulate PGC-1α expression and activity (Figure 2).

## 4 Therapeutic agents that can regulate PGC-1α activity

**FIGURE 3:**
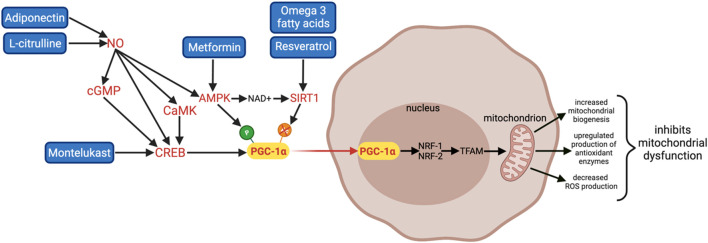
A summary of 6 promising therapies that can activate PGC-1α and its downstream pathways: Adiponectin, L-citrulline, Montelukast, Metformin, omega 3 fatty acids, and Resveratrol. Adiponectin and L-citrulline increase NO bioavailability; NO can indirectly upregulate PGC-1α through multiple pathways including the CREB, NO/cGMP/CREB, NO/CaMK pathways, and AMPK activation. Montelukast activates PGC-1α transcription directly via the CREB pathway. Metformin is an AMPK activator; it activates PGC-1α via phosphorylation. Omega 3 fatty acids and Resveratrol are SIRT1 activators; they activate PGC-1α via deacetylation. Therapies that can activate PGC-1α are beneficial in treating WMI and BPD by increasing mitochondrial biogenesis, detoxifying ROS to inhibit oxidative stress and mitochondrial dysfunction, and inhibiting inflammation.

PGC-1α can be transcriptionally regulated through the cAMP response element-binding (CREB) pathway and activated by post-translational modifications including deacetylation by sirtuin 1 (SIRT1) and phosphorylation by adenosine monophosphate-activated kinase (AMPK) (Figure 3). AMPK can also indirectly activate PGC-1α through activation of SIRT1 by increasing nicotinamide adenine dinucleotide (NAD+) levels. Upon activation, PGC-1α translocates from the cytoplasm to the nucleus where it co-activates the transcription of multiple transcription factors including NRF-1 and NRF-2. NRF-1 and NRF-2 are transcription factors for TFAM, which regulates aspects of energy metabolism including synthesis of ETC subunits and mitochondrial biogenesis.

Moreover, mitochondrial function and biogenesis are also regulated by complex mechanisms where nitric oxide (NO) is a key factor (Figure 3). NO modulates the expression of PGC-1α and its downstream effectors through four different pathways: increasing the second messenger cyclic guanosine monophosphate (cGMP) via activation of particulate guanylyl cyclase, activating AMPK, upregulating CREB, and via the calcium–calmodulin-dependent protein kinase (CaMK), an upstream activator of CREB (Riccio et al., 2006; Lira et al., 2010; Fernandez-Marcos and Auwerx, 2011; Rius-Pérez et al., 2020). Hence, therapies that increase NO bioavailability can also enhance PGC-1α activity.

PGC-1α expression and activation are complex and extensively controlled so while we discuss AMPK and SIRT1 activators, the CREB pathway, and NO donors, it is important to note that there are numerous transcriptional, post-transcriptional, and post-translational modifications to consider and explore (Martínez-Redondo et al., 2015; Miller et al., 2019; Jannig et al., 2022). Notably, while PGC-1α modulation may be one potential mechanism by which these therapeutics act, their efficacy is likely attributed to the modulation of numerous pathways. Furthermore, although increased PGC-1α gene and protein expression may be beneficial, it does not necessitate increased PGC-1α activity and further, an effect on the downstream pathways regulated by PGC-1α. Current studies show promising results however, further studies are critical in understanding how the discussed therapeutics have favorable effects.

### 4.1 AMPK activators

#### 4.1.1 Metformin

Metformin is an ancient traditional herbal medicine naturally found in the French lilac flower now commonly used to treat diabetes. It can upregulate PGC-1α via AMPK phosphorylation. There is extensive evidence to support metformin as an AMPK activator in skeletal muscle, liver, heart, stomach, pancreas, and kidney (Miao and Scutt, 2002; Suwa et al., 2006; Kristensen et al., 2013; Aatsinki et al., 2014; Chan and Rubin, 2017; Izzo et al., 2017). Metformin prevents neurodegeneration and encourages neural repair in pre-clinical studies of childhood brain injury and neonatal stroke in rodents (Dadwal et al., 2015; Kang et al., 2017; Rotermund et al., 2018; Ruddy et al., 2019). It is also helpful in treating COVID-related lung inflammation and acute respiratory distress syndrome (ARDS) (Xian et al., 2021). Metformin attenuates inflammation and prevents the onset of ARDS by decreasing IL-1β and IL-6 mRNA and protein production, and NLRP3 inflammasome activation in mice (Xian et al., 2021). Additionally, it alleviates pulmonary fibrosis and prevents PH in pre-clinical studies (Agard et al., 2009; Chan and Rubin, 2017; Goncharov et al., 2018; Liu et al., 2019a; Mulkareddy and Simon, 2020; Cheng et al., 2021; Wu et al., 2021).

Metformin is currently one of few oral medications approved for use in children 10 years of age or older and in mothers with gestational diabetes (Wu et al., 1999; Handschin and Spiegelman, 2006; Villena, 2015). Metformin crosses the placenta; its reported effects on infants *in utero* are mixed. Some data report no adverse effects of *in utero* metformin exposure on the fetus and normal growth and development during infancy and childhood (Glueck et al., 2004; Given et al., 2018; Landi et al., 2019; Greger et al., 2020; Jorquera et al., 2020). Whereas others showed a strong association between *in utero* metformin exposure and decreased birth weight, excessive weight gain in infancy, and higher BMI during childhood (Ijäs et al., 2015; Tarry-Adkins et al., 2019; Estrella et al., 2021; Yu et al., 2021). These findings are concerning since low birth weight followed by rapid postnatal growth puts infants at a higher risk of developing cardiovascular disease and type 2 diabetes (Nguyen et al., 2018; Tarry-Adkins et al., 2019; Estrella et al., 2021). Thus, additional studies are necessary to determine whether metformin is a safe therapeutic to increase PGC-1α activity and target the pathological processes contributing to WMI and BPD in neonates.

### 4.2 SIRT1 activators

#### 4.2.1 Resveratrol

Resveratrol is a polyphenol compound naturally found in red grapes, berries, peanuts, and cocoa. It has potent antioxidant, anti-inflammatory, and anti-apoptotic properties, and activates PGC-1α through the SIRT1 pathway (Tellone et al., 2015; Hoda et al., 2017; Gomes et al., 2018; Xuefei et al., 2021; Zhou et al., 2021). The preponderance of published data, reviewed by Zhu and colleagues, supports the therapeutic benefit of resveratrol in treating respiratory diseases such as BPD-PH, asthma, and pulmonary fibrosis in neonatal and adult models (Liu et al., 2015; Xu et al., 2015; Zhu et al., 2017). Resveratrol upregulates SIRT1, PGC-1α, NRF-1, and TFAM in human alveolar epithelial cells exposed to hyperoxia, improving mitochondrial dysfunction, reducing mitochondrial ROS, and apoptosis (Zhu et al., 2021). It inhibits lipopolysaccharide (LPS)-induced activation of the NF-κB inflammatory pathway, secretion of pro-inflammatory cytokines, and the NLRP3 inflammasome in rodents (Jiang et al., 2016; Wang et al., 2017). Both resveratrol and acetylresveratrol, a prodrug or inactive form of resveratrol, reduce mortality, prevent LPS-induced morphological changes in the lung, activation of NF-κB, secretion of pro-inflammatory cytokines, as well as LPS-induced inhibition of SIRT1 expression in adult mice (Özdemir et al., 2014; Ma et al., 2015). Furthermore, resveratrol inhibits injury-induced human pulmonary microvascular endothelial cell apoptosis and PASMCs proliferation which play a fundamental role in PH pathophysiology (Xia et al., 2011; Chen et al., 2014). Resveratrol also preserves myelination and protects against neurodegeneration in a variety of neurodegenerative diseases such as Alzheimer’s, Parkinson’s, and Huntington’s disease (Xiang et al., 2011; Tellone et al., 2015). It activates PGC-1α signaling, promoting mitochondrial biogenesis, improving oxidative stress, and decreasing ROS levels in a preclinical model of subarachnoid hemorrhage (Zhou et al., 2021). Lastly, resveratrol promotes M2 microglia polarization in a pro-inflammatory mileu (Nguyen et al., 2018). Despite its therapeutic potential, resveratrol has a low bioavailability, water solubility, and chemical stability. Suitable formulations, methods of administration, and storage conditions are needed due to its sensitivity to light, temperature, and pH, and further work is required to understand the effects of resveratrol on brain and lung disease in preterm infants (Zhu et al., 2017; Shaito et al., 2020).

#### 4.2.2 Omega 3 fatty acids

Omega 3 fatty acid supplementation is associated with improved health outcomes related to brain and lung diseases across the lifespan (Newberry et al., 2016; Derbyshire, 2018; Derbyshire, 2019). The three key omega 3 fatty acids: eicosapentaenoic acid (EPA), docosahexaenoic acid (DHA), and alpha-linolenic acid (ALA), are naturally found in seafood, nuts, and different oils. Additionally, breast milk is an excellent source of omega 3 for newborn infants (Berenhauser et al., 2012; Samuel et al., 2020). The positive effect of omega 3 fatty acids is largely attributed to PGC-1α activation through the SIRT1/3 pathways (Son et al., 2021). Omega 3 fatty acids are essential for brain and retinal development during the postnatal period and decreased levels are associated with increased risk for neonatal morbidities such as respiratory disease, decline in cognitive and motor abilities, and decreased postnatal growth (Martin et al., 2011; Bernardi et al., 2012; Fink et al., 2016; Lapillonne and Moltu, 2016; Gould et al., 2018). Maternal omega 3 fatty acid supplementation protects the newborn rat brain against WMI; specifically, brain weight loss, apoptosis, and hypomyelination (Tuzun et al., 2012). However, the reported effects of omega 3 fatty acids on preterm infants with BPD are varied. Some studies report that a high dose of DHA supplementation effectively decreases the incidence of BPD (Manley et al., 2011; Zhang et al., 2014; Fink et al., 2016) whereas a meta-analysis investigating the effect of omega 3 fatty acids on BPD found no significant effect of supplementation on the incidence of BPD in preterm infants (Wang et al., 2019a). The dosage, frequency of administration, as well as pharmaceutical ingredients of the omega 3 fatty acid supplements given to infants in the different studies was not consistent, suggesting promise for future trials. Limited studies have investigated the mechanism for the protective effect of omega 3 supplementation. However, recent studies report that it rescues PGC-1α, SIRT1/3, NRF-1/2, and AMPK protein expression in nephrectomy rats, and significantly increases total mitochondrial content in rodent skeletal muscle tissue and human skeletal muscle cells (Vaughan et al., 2012; Liu et al., 2019b; Son et al., 2021). Omega 3 fatty acids are safe and well-tolerated orally in infants and children hence, further mechanistic studies exploring its effect on PGC-1α signaling in the brain and lung during development would enhance our understanding of the therapeutic potential in preterm infants.

### 4.3 CREB pathway

#### 4.3.1 Montelukast

Montelukast is a leukotriene receptor antagonist commonly used to treat asthma. Data from some preliminary studies support a therapeutic benefit in preterm infants with severe BPD while others found no efficacy in reducing moderate or severe BPD (Kim et al., 2009; Rupprecht et al., 2014). It blocks hyperoxia-induced abnormal alveolarization and septation indicative of BPD injury in the lungs of newborn mice (Chen et al., 2019; Chen et al., 2020). It decreases the inflammatory response by inhibiting NF-κB, reduces markers of oxidative stress, and inhibits hyperoxia-induced apoptosis in rodent lungs and alveolar epithelial cells (Chen et al., 2019). Montelukast increases mitochondrial biogenesis in the lung via activation of the CREB pathway, consequently upregulating the expression of the PGC-1α-NRF-1-TFAM pathway (Wang et al., 2019b). CREB is an important transcription factor that can directly regulate PGC-1α expression (Wu et al., 1999; Fernandez-Marcos and Auwerx, 2011; Schmidt and Mandrup, 2011; Cheng et al., 2012; Miller et al., 2019; McMeekin et al., 2021). Montelukast treatment significantly increases mitochondrial mass, DNA, and function including respiration and ATP production in bronchial epithelial cells (Wang et al., 2019b). CREB inhibition in these cells via the H89 pharmacological inhibitor prevents montelukast-induced PGC-1α upregulation (Wang et al., 2019b). Moreover, montelukast is neuroprotective in young children and adults with brain tumors, traumatic brain injury, brain ischemia, and epilepsy but has not been tested in infants with WMI (Biber et al., 2009; Rupprecht et al., 2014; Eriksson et al., 2018; Fatima et al., 2019; Tesfaye et al., 2021). Overall, montelukast is a low-risk drug that has been used in infants and children of various ages with no significant adverse effects reported. These promising data support future trials to determine its efficacy in reducing WMI in preterm infants (Kim et al., 2009; Hon et al., 2014; Rupprecht et al., 2014).

### 4.4 Nitric oxide donors

#### 4.4.1 L-citrulline

L-citrulline is a non-essential amino acid naturally found in watermelons. It is an intermediate in the urea cycle where L-arginine is metabolized by nitric oxide synthase (NOS) to produce L-citrulline and NO thus, serving as a NO donor (Rashid et al., 2020). Preterm infants have significantly lower L-citrulline plasma levels than neonates born at term and subsequently, decreased NO bioavailability (Contreras et al., 2017). Plasma L-citrulline levels are lower in infants with BPD-PH than those without PH, suggesting that L-citrulline may be used as a biomarker (Montgomery et al., 2016). Moreover, adults with severe sepsis who develop ARDS have lower plasma levels of L-citrulline than those who do not develop ARDS (Ware et al., 2013).

L-citrulline has been used to reduce the severity of injury in hyperoxic and inflammatory models of lung injury during development. Pre-treatment with L-citrulline prevents hyperoxia or inflammation-induced arrested alveolar and vascular growth in newborn rodent models of BPD-PH (Vadivel et al., 2010; Grisafi et al., 2012; Ma et al., 2017; Dedja et al., 2018; Lauterbach et al., 2018; Martinho et al., 2020). L-citrulline supplementation also has neuroprotective effects. It prevents neuronal death and cerebrovascular injury, and increases endothelial NOS expression in a mouse model of adult brain ischemia (Yabuki et al., 2013). The protective effects of L-citrulline in the brain and lung have largely been attributed to increased NO bioavailability as NO is critical for normal brain and lung development (Ergenekon and Gücüyener, 1998; Ricciardolo, 2003; Garthwaite, 2008; Paul and Ekambaram, 2011; Garry et al., 2015). While L-arginine supplementation has also been investigated, its efficacy in increasing NO levels is limited by the first pass effect hence, L-citrulline is a more potent NO donor (Agarwal et al., 2017; Khalaf et al., 2019; Scott et al., 2021). The importance of L-citrulline as a NO donor is undeniable however, NO treatment alone does not decrease BPD incidence in human infants and nearly 40% of infants are resistant or fail to respond to inhaled NO (Raffay et al., 2012; Martinho et al., 2020). Recently, Villareal and colleagues determined that L-citrulline increases PGC-1α mRNA and protein expression in mouse skeletal muscle via increased NO production and phosphorylation of CREB (Villareal et al., 2018). As outlined above, NO can activate PGC-1α to upregulate mitochondrial biogenesis via four different pathways: cGMP generation, AMPK activation, CREB pathway, and CaMK protein (Borniquel et al., 2006; Riccio et al., 2006; Lira et al., 2010; Fernandez-Marcos and Auwerx, 2011; Baldelli et al., 2014; Craige et al., 2016; Rius-Pérez et al., 2020). Since L-citrulline is safe, well-tolerated orally, and readily available, it is an ideal natural biologic for use in preterm neonates (Ma et al., 2017; Villareal et al., 2018; Roberts et al., 2021). Further studies are needed to determine its efficacy in preventing BPD-PH and improving neurodevelopmental outcomes directly or indirectly as infants with BPD-PH have a significantly increased risk for poor neurodevelopment outcomes (Choi et al., 2019; DeMauro, 2021; Grelli et al., 2021).

#### 4.4.2 Adiponectin

Adiponectin, secreted by adipose tissue, binds to AdipoR1 and AdipoR2 receptors on many cells throughout the body with pleiotropic effects, including vasodilatory, angiogenic, anti-inflammatory, and antioxidant properties (Wang et al., 2008; Hansen-Pupp et al., 2015; Gauda and Master, 2018). It is well known for its effects on metabolism including improved glucose tolerance, increased fatty acid oxidation in liver and skeletal muscle, and decreased vascular inflammation. Serum adiponectin levels are indirectly correlated with fat mass in adults, whereas they are directly proportional in infants (Hansen-Pupp et al., 2015; Gauda and Master, 2018). Therefore, serum adiponectin is significantly lower in preterm infants than in full-term infants (Kamoda et al., 2004; Siahanidou et al., 2007; Saito et al., 2011; Oberthuer et al., 2012; Han et al., 2021). Adiponectin crosses the blood-brain barrier and binds to AdipoR1 and AdipoR2, expressed in brain endothelial cells (Thundyil et al., 2012). It protects the brain from cerebral ischemic injury, neurodegenerative diseases such as Alzheimer’s disease, as well as psychological disorders including post-traumatic stress disorder, anxiety, and depression (Hu et al., 2015; Na et al., 2017; Zhang et al., 2017; Bloemer et al., 2018; Wu, 2019; Polito et al., 2020; Li et al., 2021). Adiponectin deficiency is associated with increased white matter lesions, increased mortality post-ischemic stroke, and decreased cognitive function in adults (Efstathiou et al., 2005; Quan et al., 2022). A study in newborn rats shows that exogenous adiponectin can attenuate hypoxia-ischemia-induced brain injury by binding to its receptors and upregulating AMPK signaling (Xu et al., 2018). Adiponectin and its receptors are also expressed in various cell types in the lungs (Garcia and Sood, 2012). It has a protective role in inflammatory lung injuries such as COPD and asthma and pre-treatment with adiponectin also prevents LPS-induced BPD phenotype in newborn rats (Garcia and Sood, 2012; Ivanovska et al., 2020). Preclinical studies using adiponectin knockout mice corroborate findings in other rodent injury models showing increased lung injury and inflammation with adiponectin deficiency (Shah et al., 2022).

The preponderance of data suggest adiponectin signaling could protect the newborn brain and lung from oxidative injury via its antioxidant and anti-inflammatory properties. Interestingly, the different pharmacological agents outlined above, resveratrol, L-citrulline, metformin, and DHA (omega 3 fatty acids) all increase plasma adiponectin levels (Adamia et al., 2007; Gray et al., 2013; Joffin et al., 2015; Jimoh et al., 2018). Importantly, adiponectin may increase mitochondrial biogenesis through indirect PGC-1α upregulation. It activates PGC-1α *via* CaMK, AMPK, and SIRT1 activation by inducing calcium influx through the AdipoR1 receptor (Iwabu et al., 2010; Guo et al., 2020). AdipoR1 activation also effectively enhances NO production, a downstream regulator of various pathways which modulate PGC-1α expression and activity (Chen et al., 2003; Goldstein et al., 2009; Cohen et al., 2022). Despite its potential, high plasma adiponectin levels and AdipoR signaling have also been associated with chronic inflammation, including autoimmune diseases, chronic kidney disease, inflammatory bowel disease, and cystic fibrosis (Choi et al., 2020). Presently, small molecules that bind to adiponectin receptors are being explored since adiponectin is not available for use in humans. These agonists show promise in adult models of disease (Iwabu et al., 2010; Okada-Iwabu et al., 2013; Balasubramanian et al., 2022).

## 5 Conclusion

Mitochondrial dysregulation occurs in many developing cells and tissues as a result of oxidative stress and inflammation that are operative in the pathogenesis of WMI and BPD in premature infants. The transcriptional coactivator PGC-1α is a dynamic and potent transcriptional coactivator and master regulator of mitochondrial biogenesis and function therefore, therapeutics that can upregulate its expression and activity may be beneficial for use in diseases with underlying mitochondrial dysfunction. Upregulating PGC-1α activity, a key regulator of cellular metabolism, is also critical in addressing the large energy demands to support postnatal organ development. While therapies including metformin, resveratrol, omega 3 fatty acids, montelukast, L-citrulline, and adiponectin show great promise, there is much to be done to better understand and uncover how these therapeutics may affect PGC-1α activity. The precise mechanisms of action are complex and warrant further research. Most existing studies investigate PGC-1α signaling in highly metabolic tissues such as skeletal, cardiac, and adipose tissue so our understanding of PGC-1α such as the isoforms present, their functions, and how they are regulated in the brain and lungs are especially limited. Future studies in this field will allow us to better understand how the therapeutics discussed may beneficially regulate PGC-1α in the brain and lungs. Importantly, further studies are needed to understand their safety and efficacy in preterm infants at risk for brain and lung injury. It is also necessary to determine the optimal dose to maximize health benefits without toxicity, short and long-term side effects, and off-target effects that would be detrimental to the developing premature infant (Kaplowitz, 2017; Yang et al., 2018; Soliman et al., 2020; Raman and Foster, 2021).
